# Surface-Modification of Carbonate Apatite Nanoparticles Enhances Delivery and Cytotoxicity of Gemcitabine and Anastrozole in Breast Cancer Cells

**DOI:** 10.3390/pharmaceutics9020021

**Published:** 2017-06-07

**Authors:** Fitya Syarifa Mozar, Ezharul Hoque Chowdhury

**Affiliations:** Advanced Engineering Platform (AEP) and Jeffrey Cheah School of Medicine and Health Sciences, Monash University Malaysia, 47500 Bandar Sunway, Malaysia; fitya.mozar@monash.edu

**Keywords:** carbonate apatite (CA), nanoparticles (NPs), intracellular delivery, breast cancer cells, hydrophobicity, hydrophilicity, cellular uptake, cytotoxicity, anastrozole, gemcitabine

## Abstract

pH sensitive nanoparticles of carbonate apatite (CA) have been proven to be effective delivery vehicles for DNA, siRNAs and proteins. More recently, conventional anti-cancer drugs, such as doxorubicin, methotrexate and cyclophosphamide have been successfully incorporated into CA for intracellular delivery to breast cancer cells. However, physical and chemical properties of drug molecules appeared to affect their interactions with CA, with hydrophillic drug so far exhibiting better binding affinity and cellular uptakes compared to hydrophobic drugs. In this study, anastrozole, a non-steroidal aromatase inhibitor which is largely hydrophobic, and gemcitabine, a hydrophilic nucleoside inhibitor were used as solubility models of chemotherapy drug. Aggregation tendency of poorly soluble drugs resulting in larger particle-drug complex size might be the main factor hindering their delivery effectiveness. For the first time, surface modification of CA with poly(ethylene glycol) (PEG) has shown promising result to drastically reduce anastrozole- loaded CA particle size, from approximately 1000 to 500 nm based on zeta sizer analysis. Besides PEG, a cell specific ligand, in this case fibronectin, was attached to the particles in order to facilitate receptor mediated endocytosis based on fibronectin–integrin interaction. High-performance liquid chromatography (HPLC) was performed to measure uptake of the drugs by breast cancer cells, revealing that surface modification increased the drug uptake, especially for the hydrophobic drug, compared to the uncoated particles and the free drug. In vitro chemosensitivity assay and in vivo tumor regression study also showed that coated apatite/drug nanoparticle complexes presented higher cytotoxicity and tumor regression effects than uncoated apatite/drug nanoparticles and free drugs, indicating that surface modification successfully created optimum particles size with the consequence of more effective uptake along with favorable pharmacokinetics of the particles.

## 1. Introduction

Breast cancer is known to be one of the most common cancers in women worldwide. With expected increase in incidence and mortality, urgent development of effective cancer treatment approaches is necessary [1]. Current standard treatments mostly consist of chemotherapy, radiotherapy and hormonal therapy, causing many side effects to the patients due to non-specific cytotoxic effects on normal cells.

Delivering cancer therapeutic agents to tumor sites remains one of the challenges in chemotherapy, which is characterized by limited effectiveness due to uneven biodistribution throughout the body with absence of specific affinity toward a pathological site (organ, tissue, or cell), and lack of sufficient specificity for a cellular target. Large doses of a drug are, therefore, required in order to achieve the high local concentration and required therapeutic activity of the drug [2,3]. Multidrug resistance (MDR) issues, largely caused by overexpression of P-glycoprotein transporters, also emerge as a major obstacle, affecting the therapeutic efficacy [4]. However, these drawbacks could be overcome by controlling drug delivery to the target site of action by utilizing nanoparticle-based drug carriers, thus minimizing the drug exposure to the non-target vital tissues and eliminating undesirable side effects [5,6].

Nanoparticle entrapment of drugs confers a plenty of benefits in drug delivery including improvement of solubility of hydrophobic drugs, tissue-targeted delivery of drugs by virtue of enhanced permeability and retention (EPR) effect, active targeting by tissue specific ligand–receptor interactions with more specific cellular uptake and concomitant reduction of toxicity to normal organs, increase in half life of the drugs by prolonging their circulation time [7], and, lastly, overcoming of multidrug resistance (MDR) by taking advantage of the endocytosis-mediated internalization and thus bypassing cell surface efflux pumps.

Inorganic nanoparticles offer several advantages over organic ones for the purpose of drug delivery. They are easier to prepare with a defined size and a very narrow size distribution. Moreover, inorganic particles could be designed to have multiple functions at once, for instance, as heat generation and contrast agents. In contrast, most organic nanoparticles serve only as drug reservoirs. Additionally, inorganic nanoparticles are more stable than organic nanostructures, which still face unresolved problems, such as their limited chemical and mechanic stability and swelling [8,9,10,11]

Among the inorganic nanocarriers, carbonate apatite with the molecular formula, Ca_10_(PO_4_)_6−x_(CO_3_)_x_(OH)_2_ presents an attractive option for drug delivery due to its biodegradability, heterogeneous charge distribution, and ability of preventing crystal growth for generation of nanoscale particles [12].

Most of the discovered drugs have very low water solubility, posing great challenges to the successful development of new drugs in the pharmaceutical industry. Major issues associated with poorly water soluble compounds are limited bioavailability, suboptimal dosing, use of harsh excipients such as toxic cosolvent or surfactant, use of extreme basic or acidic conditions to enhance solubilization, and uncontrollable precipitation after dosing [13].

Recently, various approaches have been developed in order to enhance the dissolution rate of poorly soluble drugs. Physical modifications often aim to increase the surface area and solubility, since non-polar molecules tend to aggregate with each other, creating large size particles and thus, impeding their bioavailability [14]. By reducing the particle size, the increased surface area improves the dissolution properties of the drug [15].

The solubility of poorly water-soluble drug can also be improved by various solubilizing agents such as polyethylene glycol (PEG) [16,17]. In this study, a non-covalent (biotin–streptavidin) coupling procedure for the preparation of PEGylated gemcitabine-loaded CA nanoparticles is presented. Streptavidin was used as a linker between biotinylated polyethylene glycol (PEG) and CA nanoparticles. Negatively charged streptavidin was allowed to bind CA particles having Ca^2+^-rich domains, followed by ligand–receptor binding between biotinylated PEG and streptavidin, creating biotinylated PEG–streptavidin–drug loaded CA complexes. A cell specific targeting moiety, in this case fibronectin, was also attached via ionic bonding to drug-loaded CA particles in order to enhance targeting specificity to cancer cells. 

Characterization of fibronectin-biotinylated PEG–streptavidin–drug loaded CA complexes was performed by turbidity measurement, zeta sizer analysis, in vitro cytotoxicity assay, and cellular uptakes studies to determine effectivity of surface modified CA particles as a potential targeted drug carrier. Anastrozole, a non-steroidal third generation aromatase inhibitor is largely hydrophobic, while gemcitabine, a nucleoside analog is a hydrophilic drug. In this research, we explored the impact of surface modification of CA particles on enhancement of cellular delivery and therapeutic potency of anastrozole and gemcitabine.

## 2. Methods

### 2.1. Reagents

Dulbecco’s modified Eagle medium (DMEM) was purchased from BioWhittaker (Walkersville, MD, USA). DMEM powder, foetal bovine serum (FBS) and trypsin-ethylenediamine tetraacetate (trypsin-EDTA) were obtained from Gibco BRL (Carlsbad, CA, USA). Calcium chloride dihydrate (CaCl_2_·2H_2_O), sodium bicarbonate, dimethyl sulphoxide (DMSO) and thiazolyl blue tetrazolium bromide (MTT) were from Sigma-Aldrich (St. Louis, MO, USA). Streptavidin from Streptomyces avidinii, Poly(ethylene glycol) 2-aminoethyl ether biotin, and fibronectin (derived from human plasma) were obtained from Sigma Aldrich (St. Louis, MO, USA). The chemotherapy drugs, gemcitabine and anastrozole, were purchased from Sigma Aldrich (St. Louis, MO, USA).

### 2.2. Turbidity Measurement in pH-responsive Dissolution Study

100 mL of DMEM was prepared using 1.35 g of DMEM powder and 0.37 g of sodium bicarbonate with the pH subsequently adjusted to 7.5 using 0.1 M hydrochloric acid. One hundred microliters of DMEM solution was added to each microcentrifuge tube, followed by mixing of 5 μL of 1 M calcium salt and incubation at 37 °C for 30 min. For surface modification, after 30 min incubation, 1 μL of 1 μM of streptavidin was added prior to incubation for additional 10 min, followed by addition of 1 μL of 1 μM biotinylated PEG (with the resultant ratio of streptavidin to biotin–PEG being 1:1) and 1 μL of 1 μM fibronectin in succession, each with 6 min incubation. The medium was finally topped up to 1 mL with DMEM of different pHs (7.5, 6.5, 4.5 and 3.5), and the turbidity was immediately measured using 1800 MS UV spectrophotometer at 320 nm wavelength.

### 2.3. SDS-PAGE and Silver Staining

Formation of surface-modified CA was followed by centrifugation at 13,000 rpm for 15 min with the temperature maintained at 4 °C. After the supernatant was discarded, the pellet was resuspended with 100 μL DMEM. Six microliters of samples and loading dye with 1:1 ratio were loaded into each gel well (BioRad Precast Gels 7.5%) and run through SDS-PAGE at 60 V for 1 h. The resulting gel was further processed for silver staining. The gel was fixed in fixation solution (50% methanol, 12% acetic acid, and 0.5 mL/L 37% formaldehyde) for 60 min, washed sequentially with 50% ethanol for 20 min, 20% ethanol for another 20 min and ddH20 for 2 × 20 s, sensitized with sensitizing solution (0.25 g/L sodium thiosulfate) for 1 min and again washed with ddH2O for 2 × 20 s. Then, the gel was stained with 2 g/L silver nitrate + 0.75 mL/L 37% formaldehyde for 20 min and washed with ddH20 for 2 × 2 min. Finally, after adding developing solution (60 g/L sodium carbonate, 0.5 mL/L 37% formaldehyde and 4 mg/L sodium thiosulfate), the gel was shaken gently until the bands appeared. Fifty percent of methanol + 12% acetic acid was used to stop the staining process.

### 2.4. Cell Culture and Seeding

MCF-7 and 4T1 cells were grown in 25 cm^2^ culture flask in DMEM supplemented with 10% heat-inactivated FBS in a humidified atmosphere containing 5% CO_2_ at 37 °C. Exponentially growing MCF-7 and 4T1 cells were trypsinised and following addition of fresh medium, the cell suspension was centrifuged at 120 rcf for 5 min and the supernatant was discarded. Fresh medium was added to resuspend the cell pellet and the cells were counted using a haemocytometer. Appropriate dilutions were made using culture medium to produce a cell suspension with concentration 5.0 × 10^2^ cells/mL. One milliliter of the prepared cell suspension was subsequently added into each of the wells in a 24-well plate and subjected to overnight incubation at 37 °C and 5% CO_2_ before treatment.

### 2.5. Intravenous Delivery in a 4T1-Induced Murine Breast Cancer Model

Female Balb/c mice (6–8 weeks old) of 15–20 gm of body weights (obtained from School of Medicine and Health Science animal facility, Monash University) were maintained in 12:12 light:dark condition and provided with ab libitum and water. All the experiments done were done in accordance with the protocol approved by MONASH Animal Ethics Committee (MARP/2016/126). Approximately 1 × 10^5^ 4T1 cells (in 100 µL PBS) were injected subcutaneously on the mammary pad. When the volume of the growing tumor reached an average 13.20 ± 2.51 mm^3^ at around Day 6–8, mice were grouped randomly with 6 mice per group and treated intravenously (tail-vein) at the right or left caudal vein, while the second dose was administered after 3 days of the 1st dose.

The lengths and widths of the outgrowth tumor were estimated using the vernier caliper in mm scale over the period of 22 days, with the data subsequently presented as mean ± SD of the tumor volumes of six different mice from each group. The volume of the tumor was calculated based on the following formula:

(1) $$\mathrm{Tumor}\;\mathrm{volume}\;({\mathrm{mm}}^{3})\;=\;1/2\;(\mathrm{Length}\;\times \;{\mathrm{Width}}^{2})$$

### 2.6. Cell Treatment

The next day, 100 mL of DMEM was prepared by mixing 1.35 g of DMEM powder and 0.37 g of sodium bicarbonate and adjusting the pH to 7.4 using 0.1 M hydrochloric acid. The prepared DMEM solution was filtered using 0.2 μm syringe filter in laminar flow hood, followed by transferring of 1 mL of the filtered medium into a 1.5 mL microcentrifuge tube. Drugs were added into each of the microcentrifuge tubes (from 100 pM to 1 μM concentration), followed by addition of 3 µL of CaCl_2_ and incubation at 37 °C for 30 min for generation of drug-loaded particles. For particle surface modification, 1 μL of 1 μM of streptavidin was added to the suspension of drugs/particle complexes before further incubation for 10 min, followed by addition of 1 μL of 1 μM biotinylated PEG and 1 μL of 1 μM fibronectin sequentially, with 6 min incubation for each. Ten-percent FBS was then added into each microcentrifuge tube. Culture medium from the wells with the cells seeded one day before was aspirated and replaced with 1 mL of the prepared particle suspension formulated with or without drugs. Plates were then incubated at 37 °C and 5% CO_2_ for two consecutive days.

### 2.7. Cell Viability Assessment with 3-(4,5-dimethylthiazol-2-yl)-2,5-diphenyltetrazolium Bromide (MTT) Assay

Following two days of treatment, the fraction of viable cells was determined using MTT assay. Briefly, 50 μL of MTT (5 mg/mL in PBS) was added aseptically into each of the wells in the plates, followed by incubation at 37 °C and 5% CO_2_ for 4 h. After the incubation, medium containing MTT was aspirated and the purple formazan crystals at the bottom of each well were dissolved by mixing with 300 μL of DMSO solution. Absorbance of the resulting formazan solution was then determined spectrophotometrically at wavelength 595 nm using microplate reader (Dynex Opsys MR, Chantilly, VA, USA) with reference to 630 nm. Each experiment was performed in triplicates and the data were plotted as mean ± standard deviation (S.D.) of three independent experiments.

### 2.8. Data Analysis

The cell viability in the treated wells was expressed as a percentage and was calculated using the absorbance values obtained from MTT assay by using the following formula:

(2) $$\%\mathrm{cell}\;\mathrm{viability}=\frac{\mathrm{Absorbance}\;\mathrm{of}\;\mathrm{treated}\;\mathrm{sample}}{\mathrm{Absorbance}\;\mathrm{of}\;\mathrm{control}}\times 100\%$$

On the other hand, enhancement of cytotoxicity was calculated using the following formula:

Enhancement of cytotoxicity (%) = *U* – *T* (3)

where *U* is cytoxicity percentage of nanoparticle–drug complex or coated nanoparticle–drug complex with nanoparticle-treated cells as positive control, and *T* denotes cytoxicity percentage of free drugs with untreated cells as positive control. Each experiment was done in triplicates and shown as mean ± SD.

### 2.9. Estimation of Drug Binding Affinity for Particles

DMEM solution was prepared according to the protocol described above. Following addition of drugs at 20, 60 and 100 μM concentrations and Ca^2+^ at 7 mM into 1 mL of the prepared DMEM, the mixture was subjected to incubation at 37 °C for 30 min. After that, particle suspensions were centrifuged with 1400 rpm for 20 min at 4 °C. The amount of drugs (gemcitabine and anastrozole) present in the supernatant (representing the unbound drugs) was checked by HPLC (Agilent) attached with Agilent chemostation software, using zorbax SB C18 column (4.6 mm × 150 mm, 5 μm, Agilent). The conditions of HPLC set for gemcitabine, and anastrozole are summarized in Table 1 and Table 2. Different concentrations of drugs were dissolved in their solvents and run on HPLC for making standard curves by concentrations vs. peak areas.

The concentrations of the drugs present in the supernatant were calculated from the peak area, using the standard curves. Data were represented as percent of interaction efficiency of drugs with nanoparticles, calculated by using the following formula:

(4) $$\%\;\mathrm{Binding}\;\mathrm{efficiency}=\frac{\mathrm{NP}-\mathrm{bound}\;\mathrm{drugs}\;\times 100\%}{\mathrm{Total}\;\mathrm{drugs}}$$

where “NP-bound drugs” is the amount of drugs bound to the nanoparticles, which was calculated by subtracting the amount of drugs found in the supernatant from the amount of drugs initially added to form drug-nanoparticle complex, whereas “total drugs” represents the amount of drugs initially used for complexation, i.e., free and particle-bound drugs altogether. Each of the experiments was done in triplicate and shown as mean ± SD.

### 2.10. Dose-Dependent Cellular Uptake of Free and Nanoparticle-Bound Forms of Drugs

After treatment of MCF-7 cells with free drug (20 µM) and drug-loaded CA particles (formed with 20 µM of the drugs) for 1, 4 and 24 h, the culture medium was taken into 1.5 mL micro centrifuge tubes and centrifuged with 1400 rpm for 20 min at 4 °C. The concentrations of the drug present in the supernatants (unbound drug) were checked by HPLC and calculated from the peak area using the standard curves. The cellular uptake was determined based on the following formula:

Cellular uptake: Initial drug concentration–unbound drug concentration. Each experiment was done in duplicates and shown as means ± SD.

### 2.11. Particle Size and Zeta Potential Measurement

DMEM solution was prepared according to the protocol described above. Drugs at 1 µM were added to 1 mL of DMEM solustions, followed by addition of 3 mM Ca^2+^ in order for generation of drug-loaded CA particles through incubation at 37 °C for 30 min. Drug-free particles were also fabricated in a similar way in absence of the drug. After the incubation, 100 µL of FBS was added to all samples. While waiting for measurement, all samples were kept inside 4 °C chiller. Size and zeta potential measurement were done using Malvern nano zeta sizer, with attached zeta sizer software for data analysis.

Ordinary one-way ANOVA was performed using GraphPad Prism 7 to analyze surface modified-CA cell viability results, whereas Dunnett’s test for two way ANOVA was implemented to compare treatment groups (CA loaded drugs and PEGylated CA loaded drugs) and control group (free drugs) in both in vitro and in vivo studies. Values were significant (*) at *p* value 0.01 to 0.05, very significant (**) at *p* value 0.001 to 0.01, extremely significant (***) at *p* value 0.0001 to 0.001, and extremely significant (****) at *p* value < 0.0001.

## 3. Results

### 3.1. Formulation of CA Particles and Effect of Surface Modification on Their pH-sensitivity

**Figure 1:**
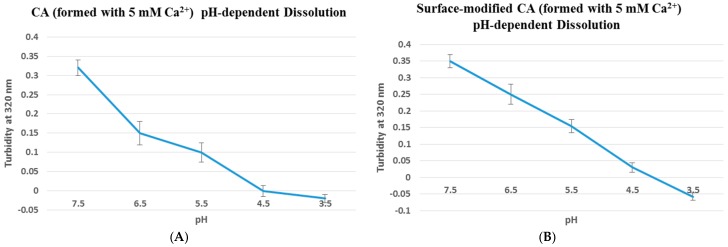
pH dissolution test was done using UV spectrophotometer at 320 nm wavelength. Particles without (**A**) or with (**B**) surface-coating (streptavidin–biotin PEG–fibronectin) were initially fabricated in 100 µL of bicarbonate-buffered DMEM media (pH 7.5), followed by addition of DMEM media of decreasing pHs (6.5, 5.5, 4.5 and 3.5) to top up to 1 mL, prior to the turbidity measurement. Turbidity value of 0.36 indicates that particles were already formed at pH 7.5, and with decreasing pHs the particles started to dissolve, resulting in lower turbidity value until reaching close to 0 where all particles were completely dissolved.

In order to optimize the formulation of CA particles, different concentration of Ca^2+^ (1–5 mM) was added to 1 mL or 100 µL of prepared DMEM, followed by incubation for 30 min at 37 °C and addition of 10% FBS (after volume adjustment to 1 mL with DMEM in the latter case). Incubation of the differently formulated particles with MCF-7 cells for 4 h and visualization of the cells under an optimal microscope showed an increasing number of aggregated particles (in both cases) with increasing Ca^2+^ concentrations (Figure not shown here), in agreement with our earlier report (12). One fascinating property of CA is its rapid dissolution after cellular internalization via endocytosis, leading to intracellular release of drugs from the carrier. To test whether surface coating with streptavidin and biotin–PEG results in reduction of acid solubility of CA particles, turbidity values of unmodified and surface-modified particles were measured at different pHs ranging from 7.5 to 3.5 (Figure 1). Both modified and unmodified CA particles were completely dissolved at pH 4 which is similar to endosomal acidic pH, demonstrating retention of pH-sensitivity property by surface-modified CA particles. However, coating density might be an important factor in determining whether protons could penetrate across the surface coat to dissolve the particles.

### 3.2. Influence of Surface Modification on Size and Surface Charge of Drug-Loaded CA Particles

**Figure 2:**
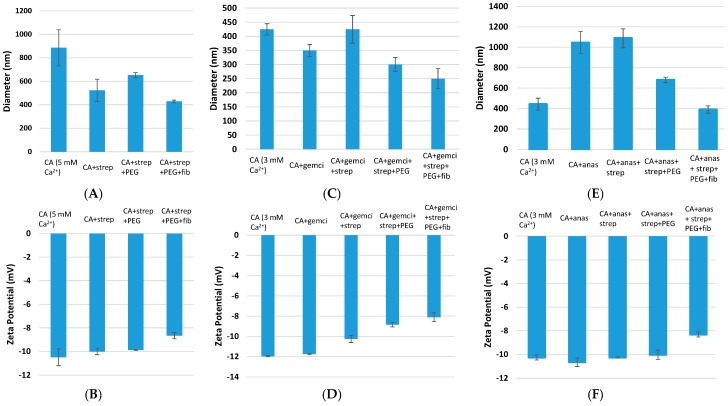
Size and zeta potential measurement of surface-modified, drug-loaded CA particles. CA (fabricated with 3 mM Ca^2+^) and CA + drug (1 μM drug concentration) were used as controls. Surface modifications included: CA + drug + streptavidin, CA + drug + streptavidin + biotin–PEG, and CA + drug + streptavidin + biotin–PEG + fibronectin. CA: carbonate apatite; strep: streptavidin; fib: fibronectin; gemci: gemcitabine; anas: anastrozole. (**A**) and (**B**) denote particle diameter and surface charge measurements for unmodified and surface-modified particles without incorporated drugs, (**C**) and (**D**) denote particle diameter and surface charge measurements for unmodified and surface-modified particles with incorporated gemcitabine, and (**E**) and (**F**) denote particle diameter and surface charge measurements for unmodified and surface-modified particles with incorporated anastrozole.

Size and zeta potential of surface-modified CA and surface-modified, drug-embedded CA were measured following fabrication of the particles with 1 μM drug concentration and constant amount of exogenous Ca^2+^ (3 or 5 mM). While the average diameter of the unloaded CA particles prepared with 5 mM of Ca^2+^ was 820 nm, the surface-modified CA ones showed a decreasing pattern of particle diameters reaching 610.5 nm after PEGylation, and 402 nm after PEGylation + fibronectin coating, respectively (Figure 2A). On the other hand, the zeta potential of the surface-modified particles shows little change compared to the particles alone, turning out to be slightly more electropositive after the modification (Figure 2B).

For gemcitabine-loaded CA particles prepared with 3 mM of Ca^2+^, PEGylation demonstrated slight size reduction from initial 340 nm average diameter to 285.4 nm (Figure 2C). Further decrease in particles size reaching ± 220 nm was achieved after addition of fibronectin coating. Zeta potential also slightly increase from −12 to −8 mV after the coating (Figure 2D).

PEGylation appeared to give a significant size reduction for anastrozole-apatite particle complex from initial 1000 nm average diameter at 1 μM drug concentration to 621.4 nm diameter, whereas addition of fibronectin coating further decreased the size to 410 nm (Figure 2E). The zeta potential slightly increased from −10 to −8 mV with PEGylation and fibronectin coating (Figure 2F).

**Figure 3:**
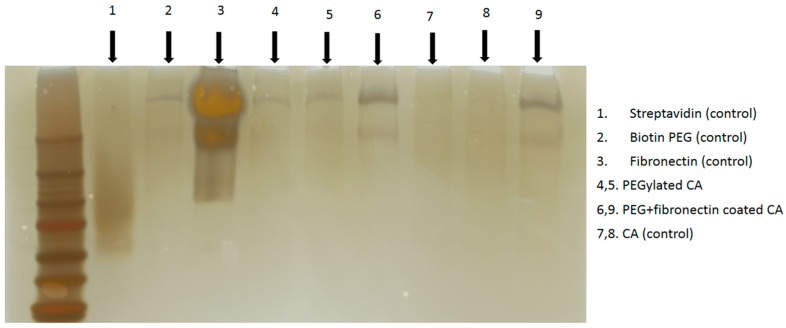
After formation of surface-modified CA as described above, centrifugation was done at 4 °C with 13,000 rpm for 15 min. The supernatant was discarded and the pellet was resuspended at 100 μL DMEM. Six microliters of samples and loading dye with 1:1 ratio were loaded into each gel well (BioRad Precast Gels 7.5%) and run through SDS PAGE at 60 V for 1 h. The resulting gel was processed through silver staining.

In order to confirm binding of streptavidin and fibronectin to the CA nanoparticles, we carried our SDS-PAGE and silver staining after formulation of surface-modified complexes. As shown in Figure 3, both biotin–PEG and fibronectin interacted with the nanoparticles, although the signal for streptavidin was not at the detectable level. Therefore, the direct binding of biotin–PEG with nanoparticle surface via electrostatic interactions could not be ruled out, since biotin moiety possesses protanable amine groups and ionizable carboxyl group.

### 3.3. Cytotoxicity Analysis for Surface-Modified, Drug-Loaded Nanoparticles

**Figure 4:**
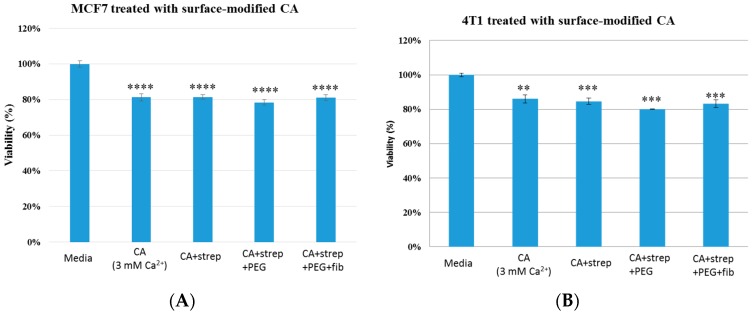
Cell viability of MCF7 (**A**) and 4T1 (**B**) cells after 48 h of treatment with CA and surface- modified CA particles. Surface modifications includes CA + streptavidin, CA + streptavidin + biotin–PEG and CA + streptavidin + biotin–PEG + fibronectin with untreated cells and CA particles alone considered as controls. CA: carbonate apatite; strep: streptavidin; fib: fibronectin.

**Figure 5:**
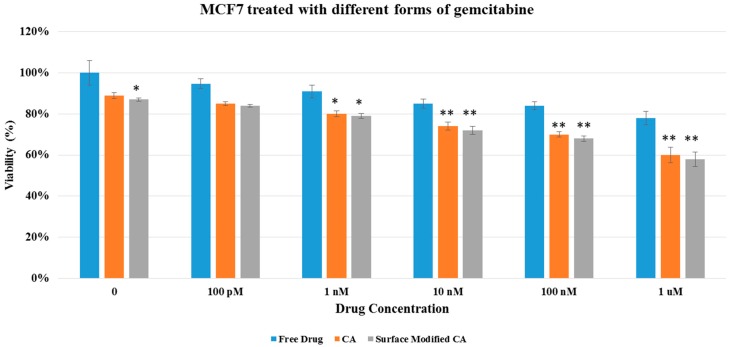
Cell viability of MCF7 after 48 h of treatment with gemcitabine-loaded CA and surface modified, gemcitabine-loaded CA. Surface-modified CA denotes the particles modified with streptavidin–biotin–PEG and fibronectin. Values were significant (*) at *p* value 0.01 to 0.05 and very significant (**) at *p* value 0.001 to 0.01.

**Figure 6:**
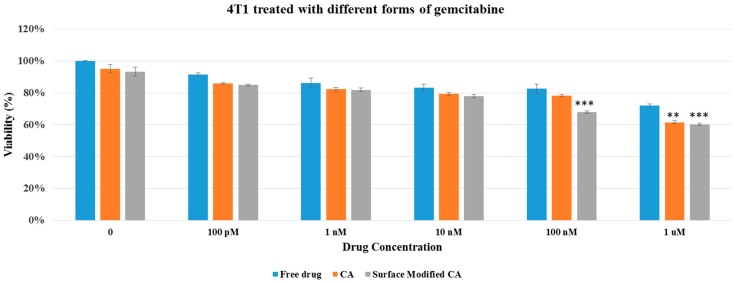
Cell viability of 4T1 after 48 h of treatment with gemcitabine-loaded CA and surface modified, gemcitabine-loaded CA. Surface-modified CA denotes the particles modified with streptavidin–biotin–PEG and fibronectin. Values were very significant (**) at *p* value 0.001 to 0.01, and extremely significant (***) at *p* value 0.0001 to 0.001.

**Figure 7:**
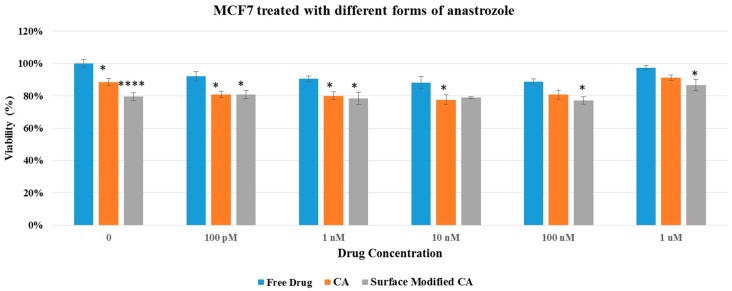
Cell viability of MCF7 after 48 h of treatment with anastrozole-loaded CA and surface modified, gemcitabine-loaded CA. Surface-modified CA denotes the particles modified with streptavidin–biotin–PEG and fibronectin. Values were significant (*) at *p* value 0.01 to 0.05, very significant (**) at *p* value 0.001 to 0.01, extremely significant (***) at *p* value 0.0001 to 0.001, and extremely significant (****) at *p* value < 0.0001.

**Figure 8:**
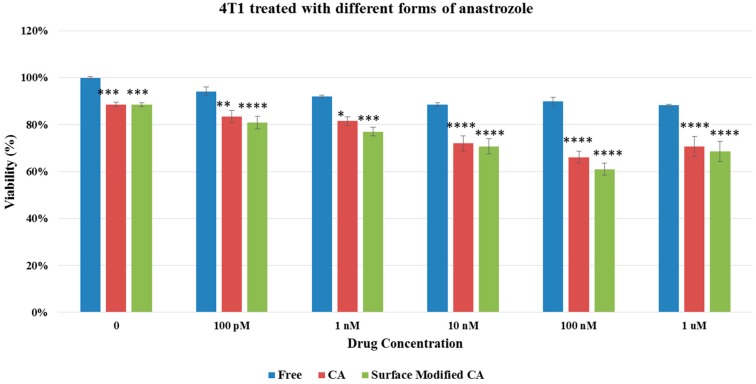
Cell viability of 4T1 cells after 48 h of treatment with anastrozole-loaded CA and surface modified, gemcitabine-loaded CA. Surface-modified CA denotes the particles modified with streptavidin–biotin–PEG and fibronectin. Values were significant (*) at *p* value 0.01 to 0.05, very significant (**) at *p* value 0.001 to 0.01, extremely significant (***) at *p* value 0.0001 to 0.001, and extremely significant (****) at *p* value < 0.0001.

Cell viability results in both MCF7 and 4T1 displayed no apparent differences between surface-modified CA and unmodified CA particles, indicating that surface modification by itself did not add toxicity to the cells (Figure 4), inspiring us to perform cytotoxicity profiling for drug-loaded, surface-modified particles. As shown in Figure 5 and Figure 6, both surface-modified and unmodified gemcitabine-loaded particles induced more cytotoxicity particularly at higher drug concentrations compared to free drugs in MCF-7 as well as in 4T1 cells. However, in terms of cytotoxicity enhancement, surface-modified particles statistically outperformed the unmodified ones at 100 nM drug concentration only in 4T1 cells (Table 3 and Table 4). Similar pattern in cytotoxicity was noticed in both cell lines with anastrozole-loaded, surface-modified particles (Figure 7 and Figure 8). In addition, similar to gemcitabine, anastrozole, when bound to the particles with surface coating, produced more cytotoxic effect than unmodified nanoparticles at 100 nM drug concentration (Table 5 and Table 6).

### 3.4. Drug Binding Affinity to CA Particles

**Figure 9:**
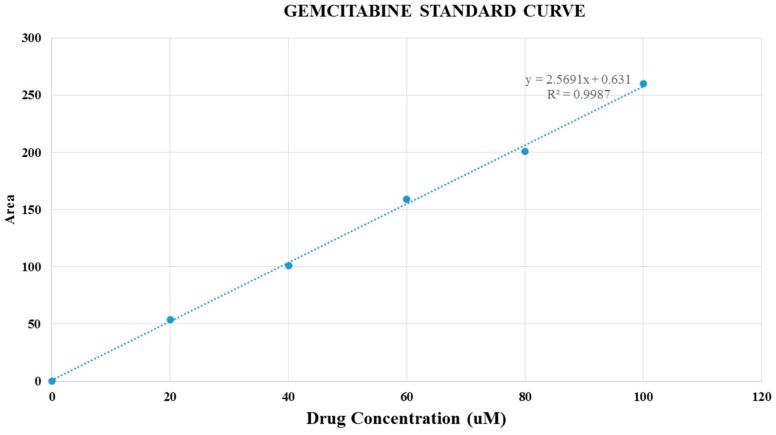
Gemcitabine standard curve.

**Figure 10:**
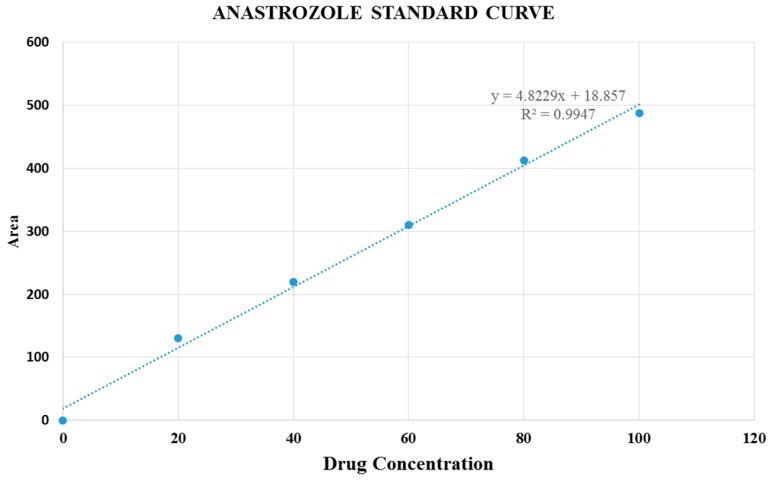
Standard curve of anastrozole.

In order to determine the affinity of drugs to CA, the calibration curve of each drug was constructed by plotting the peak area verses the known concentrations of gemcitabine (Figure 9) and anastrozole (Figure 10) introduced into the mobile phase for HPLC. The amount of the drug bound to CA particles was determined by separating the drug-loaded particles from DMEM through centrifugation and measuring the unbound drugs available in the supernatant. The result with three different drug concentrations, 20, 60, and 100 μM and constant amount of Ca^2+^ (7 mM) initially used to prepare the drug-bound particles demonstrates that the amount of gemcitabine adsorbed into the CA particles increased with higher initial drug concentrations, but remained similar in terms of percentage binding (Table 7). HPLC findings also indicated that CA binding affinity towards anastrozole was much lower compared to gemcitabine.

### 3.5. Cellular Uptake of Drugs Carried by Surface-Modified Particles

Our previous paper [12] showed that particle-mediated drug uptake was significant even at 45 min and increased further at 90 min after the treatment. In the current study, there was an apparent increase in the cellular uptakes of the drugs (1, 4 and 24 h) carried by surface-modified particles compared to free drug and the drugs delivered by unmodified particles, indicating that cell specific targeting and PEGylation facilitated more particle internalization by the cells. Longer treatment time also increased the uptake until reaching almost 100% at 24 h. Varying concentrations of Ca^2+^ impact the particles uptake efficiency depending on the incubation period, since higher Ca^2+^ concentration induces more particles growth and generation of large particles which are less effectively endocytosed than small particles. However, gemcitabine tendency to reduce particles size after incorporation might be a factor that enabled higher uptake even with higher Ca^2+^ salt concentration, as seen with both unmodified and surface-modified CA particles formulated with 9 mM of Ca^2+^, that facilitated more drug uptake at 1 h time point compared to free drugs (Table 8). At 4 h, surface-modified particles formed with 7 mM of Ca^2+^ showed higher cellular uptake than the unmodified particles formulated with the same Ca^2+^ concentration, and the free drugs as well, which might be due to the influence of surface coating in regulating particle size and specific binding to the cell membrane (Table 8).

On the other hand, cellular uptake of anastrozole bound to the unmodified and surface-modified particles was found to be more significantly enhanced compared to the free drug at 1 h and 4 h, when the particles were fabricated with 7 mM of Ca^2+^ instead of higher Ca^2+^ concentrations, shedding light on the fact that that unlike gemcitabine, anastrozole has no role in inhibiting particle growth which hampers the cellular uptake. Interestingly, surface-modified particles played a more powerful role in accelerating the drug uptake compared to the unmodified particles at those two time points, which could be due to the influences of reduced particles size as a result of surface coating in agreement with our earlier finding (Figure 2E) and integrin-mediated, specific cellular uptake for the fibronectin-coated particles.

### 3.6. Intravenous Delivery of PEGylated, Gemcitabine-Loaded CA Nanoparticles in a 4T1-Induced Murine Breast Cancer Model

**Figure 11:**
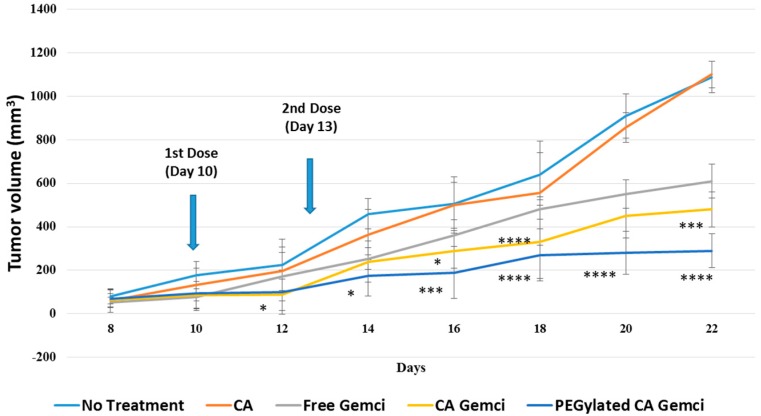
Tumor regression study following intravenous delivery of gemcitabine-loaded nanoparticles to 4T1-induced breast tumors in mice. 4T1 cells were inoculated subcutaneously on the mammary pad of mice. Tumor-bearing mice were treated intravenously through tail vein injection with 100 µL of free gemcitabine (0.5 mg/kg) and surface-modified or unmodified nanoparticles (formed with 0.5 mg gemcitabine/kg), when the tumor volume reached to 13.20 ± 2.51 mm^3^. Six mice per group were used and data were represented as mean ± SD of tumor volume. Values were significant (*) at *p* value 0.01 to 0.05, very significant (**) at *p* value 0.001 to 0.01, extremely significant (***) at *p* value 0.0001 to 0.001, and extremely significant (****) at *p* value < 0.0001.

Both surface-modified and unmodified gemcitabine-loaded CA nanoparticles were intravenously administered twice within an interval of 3 days to a 4T1 cancer cells-induced syngeneic mouse model of breast cancer. As shown in Figure 11, compared to free gemcitabine (0.5 mg/kg of a mouse), unmodified nanoparticles loaded with the same amount of the drug led to a significant reduction in tumor volume, while the surface-modified ones dramatically regressed the tumor growth, indicating that surface-modification might confer the favorable pharmacokinetics of the drug-loaded nanoparticles with higher accumulation and uptake by the tumor.

## 4. Discussion

### 4.1. Interaction of Drugs with CA Particles and Estimation of Drug Binding Affinity

Drugs interact with CA via ionic bonding at physiological pH (7.4) which triggers protonation or deprotonation of the drug ionizable groups depending on their p*K*a values to become positively or negatively charged, thus enabling their binding to the anion and cation binding domains, represented by Ca^2+^- and PO_4_^3−^/CO_3_^2−^-rich sites of the particles, respectively.

Since interaction between drugs and CA are based on ionic interaction which is a weak non-covalent, centrifugation force could easily break these bonds. In case of anastrozole which is a poorly water soluble drug, presence of DMSO solvent could a create hydrophobic layer on each of the particles, inducing their aggregation via hydrophobic-hydrophobic interactions and hence, hindering drug binding to the particles (Table 7). Moreover, particle aggregation could reduce the total surface area with eventual poor drug binding. On the other hand, gemcitabine, being water solvent, results in more stable binding to CA particles than anastrozole.

### 4.2. Surface Modification of Drug-Loaded CA Particles

The ligand binding proteins provide exceptional systems for controlled and targeted delivery of therapeutic molecules. They function by binding small molecules drug and peptides with high specificity and affinity. There are many classes of ligand binding proteins that have been used, such as biotin binding proteins, lipid binding proteins, periplasmic binding proteins, lectins etc [18,19].

**Figure 12:**
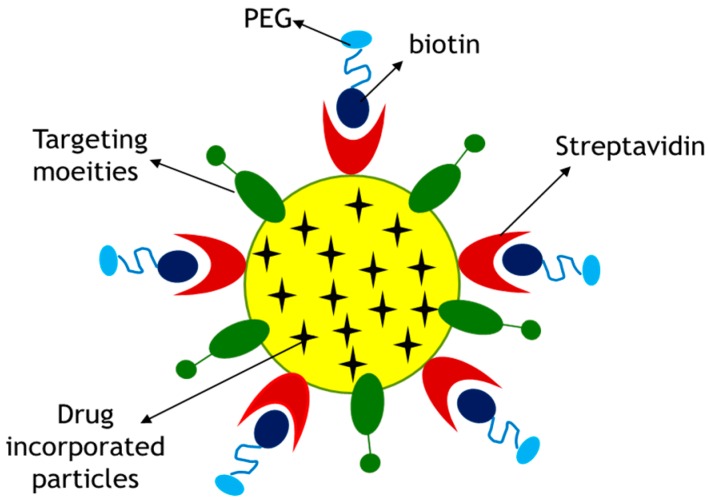
Proposed surface modification model for a drug-loaded CA particle. Surface modification involves PEGylation and attachment of a targeting moiety to drug–apatite complexes. Streptavidin was used as a linker between drug–apatite particles and biotin–PEG, since biotin has specific affinity towards straptavidin. A cell-targeting moiety, such as fibronectin, is attached to the particles via ionic interactions.

**Figure 13:**
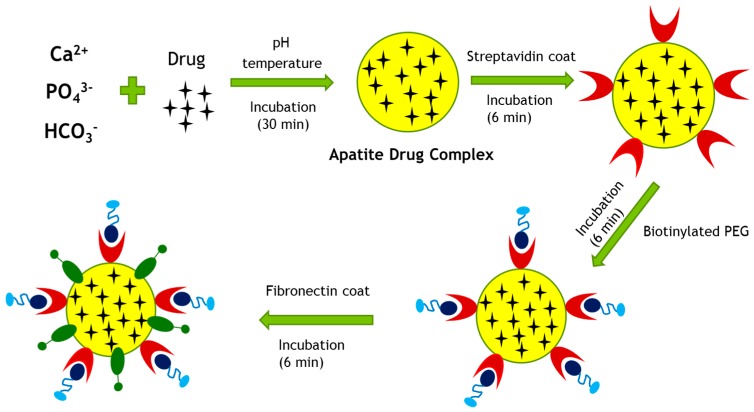
Fabrication of surface-modified, drug-loaded CA particles. The particles were fabricated by allowing interactions among the salts of Ca^2+^, inorganic phosphate and bicarbonate in DMEM with its pH adjusted to 7.4, and incubating the resulting mixture at 37 °C for 30 min. Once the apatite–drug complex were formed, streptavidin, biotinylated PEG and fibronectin were added sequentially, with 6 min incubation at 37 °C maintained for each of the steps.

In the present study, hydrophilic surface coating of drug-loaded CA particles by PEG and a cell specific ligand, fibronectin was designed to increase targeting specificity and delivery efficiency of the particle drug complex into breast cancer cells (Figure 12). A non-covalent (biotin–streptavidin) coupling procedure for the preparation of PEGylated drug-loaded CA particles was followed (Figure 13), since PEG, being highly hydrophilic and electrically neutral, is unable to form ionic bonding with CA particles. Streptavidin which carries net negative charges with potential ability to bind to Ca^2+^-rich domains of the particles was used as a linker between biotinylated PEG and drug-loaded CA complexes [20].

Hydrophobic drug–particle complexes are more quickly opsonized than hydrophilic ones [21]. In order to reduce the hydrophobic interaction with plasma proteins and the mononuclear phagocytic system (MPS) clearance, hydrophilic shielding, such as PEGylation was introduced [22,23]. However, due to steric hindrance caused by PEG chains, nanoparticle interactions with cellular membranes and endosomal membranes were reduced. Thus, a cell specific targeting moiety, such as fibronectin is necessary in order to ease cellular internalization of the PEGylated particles by fibronectin–integrin interaction, resulting in receptor-mediated endocytosis.

Physical properties of a nanocarrier, such as size, shape, and surface charges can affect its bioavailability and biodistribution. All of these factors are important for targeting purposes as well as for avoiding rapid particle uptake by MPS of the lung, liver, spleen, and bone marrow. Particles of small size (10–20 nm) tend to have wide distribution due to its ability to cross the tight junctions between the endothelial cells of blood vessels and have rapid renal clearance owing to their filtration capability through the glomeruli of kidney, while large particles (>1 μm) are subjected to rapid clearance by the macrophages of MPS and thus accumulated mainly in liver, spleen, and bone marrow. Thus, particles with size between 20 nm and 1 μm are considered ideal since they have more prolonged circulation time and also less sensitive to the clearance by kidney and MPS [24].

Solubility of the drugs plays an important role in its bioavailability inside the body. While hydrophobic drugs have easier cellular internalization compared to hydrophilic drugs due to the ability of the former to cross the lipid bilayer of cell membrane, they encounter more hindrance in blood transportation. In the circulation, blood plasma protein (fibrinogen, complement C3 and IgG) would bind to these hydrophobic drug particles, resulting in opsonin mediated endocytosis before clearance in the MPS systems [24]. Hydrophobic drug particles also have tendency to aggregate with each other creating large size particles which are not effectively internalized by the cells and more prone to clearance. Various attempts were directed towards altering the surface properties of nanoparticles to reduce their RES clearance. To generate physically stable nanoparticles, various excipients are used in order to dampen or sensitize the surface energy of the nanoparticles by way of steric and/or ionic stabilization. One of the options is by PEGylation which based on our results reduced the negative potential of the CA particle surface. Moreover, as mentioned by Petra K et al. and Porter Cl et al. in previous sutides, particles with neutral surface charges seem to be the most appropriate with regard to blood persistence [25,26].

Avidin originally has four binding sites for biotin with each pair located at opposite sides of the tetrameric structure of avidin. However, the stoichiometry between avidin and biotin–PEG might change depending on the biotin–PEG molecular weight and chain length. Previous research reported that with biotin PEG (5000), as used in this study, the measured stoichiometry was 1:1 between biotin–PEG and avidin, indicating that steric hindrance might cause PEGylated biotin of 5000 g/mol to access only one site on avidin. Meanwhile, lower chain biotin–PEG (3400) and biotin–PEG (588) both have 4:1 stoichiometry with avidin [27]. Longer chain PEG was used in this study to increase stability as well as to enhance anti-opsonisation effect of PEG for in vivo application.

PEG conjugation increases surface hydrophilicity of particles for providing their prolonged circulation in blood, better biocompability and reduced opsonization by RES. In addition, targeting moieties, such as ligands or antibodies are used to help cellular internalization of particles by receptor mediated endocytosis (active targeting), in addition to their passive accumulation (depending on their size) through leaky vasculature by the EPR effect. PEG decreases the amount of attraction between NPs by increasing the steric distance between them and increasing hydrophilicity by forming hydrogen bonds with aqueous solvent. In addition, PEG is also able to reduce opsonization process, thus preventing recognition by monocytes and macrophages and allowing the nanoparticles to remain in the circulation [28,29,30].

### 4.3. Cytotoxicity Assessment of Surface-Modified and Unmodified Drug-Apatite Complexes in MCF7 and 4T1

We showed here that intracellular uptake of gemcitabine in either free form or particle-bound form reached the maximum level (100%) with 24 h of incubation with the cells (Table 8). In addition, unlike free drugs, which enter by passive diffusion, nanoparticle-bound drugs enter by endocytosis, followed by drug release from the particles through particle dissolution at endosomal acidic pH. This process is likely a time-consuming process unlike the passive diffusion of free drugs. We carried our another study [31] which indicated that the length of incubation period had a significant effect on cytotoxicity, with 48 h incubation of cells with NP-bound drugs and free drugs resulting in more significant cytotoxicity for NP–drugs than free drugs, compared to the shorter exposure periods (4 or 24 h). In addition to the incubation time, number of cells seeded in a well of a dish also influences cell viability, with longer drug exposure time and lower number of cells eventually leading to higher cytotoxicity. Thus, 48 h incubation period was chosen in our study by considering the number of cells seeded, the amount of drugs used, and the time required for intracellular release of drug from the particles and to induce cell death with the free or released drugs. Cytotoxicity assay of drug-loaded particles in MCF- 7 and 4T1 cell lines revealed that the surface-modified drug/CA particle complexes generally exhibited higher cytotoxicity compared to unmodified drug/CA complexes and free drugs (Figure 5, Figure 6, Figure 7 and Figure 8). Surface-modified, drug-loaded apatite complexes formed with lower drug concentration displayed almost the same level of cytotoxicity as observed with higher concentration of drugs either in free form or associated with unmodified CA particles, indicating that a lower initial dose of drugs would suffice to produce the similar therapeutic effect while additionally reducing the off-target effects of drugs in other organs. The enhanced cytotoxicity of the coated drug/particle complexes could be explained by the notion that PEGylation reduced the complex size into a more optimum level, while fibronectin ligand assisted receptor-mediated endocytosis via fibronectin–integrin interaction, ultimately resulting in better cellular uptake of the drugs. Fibronectin-specific integrin was overexpressed by various cancers, including breast carcinoma. We showed in our earlier studies that fibronectin dramatically reduced the particle diameter of CA particles and enhanced selective uptake of the nanoparticles by cancer cells [32,33].

### 4.4. Role of Surface-Modified CA Particles in Time-Dependent Uptake of Gemcitabine and Anastrozole in MCF-7 Cells

Since labeling of drug molecules might alter the cell membrane permeability property of the drugs, we collected the cell culture media at different time points of cell incubation in presence of free drugs and CA-loaded drugs and analysed by HPLC to quantitate the amount of the drugs not delivered into the cells and thus the amount of the drugs taken up by the cells. The role of particles in promoting cellular uptake of the drug was also evaluated in MCF-7 cells. As shown in Table 8 and Table 9, at 20 μM initial drug concentration available in free form or allowed to complex with the particles, an enhanced uptake of the particle-associated drugs compared to the free drug was noticed at 1 and 4 h of the incubation period while at 24 h both free and particle-bound drug could accumulate almost to the similar and highest extent into the cells. A significant difference in enhancement of cellular uptake between unmodified and surface modified CA was observed in anastrozole-loaded CA particles compared to gemcitabine-loaded particles, indicating that coating might enhance more cellular uptake of the drug by decreasing the complex diameter and promoting fibronectin–integrin interaction. Given that the affinity of anastrozole for CA particles are quite low (Table 7), it can not be ruled out that the drug could even be internalized along with particles without being associated with the latter. In contrast, gemcitabine by itself gradually decreased the size of apatite/drug complexes with higher drug concentration, suggesting that the drug might be capable of limiting the particle growth [12]. As a result, surface modification apparently conferred less impact to hydrophilic drug compared to poorly water-soluble drug.

### 4.5. Tumor Regression Folowing Intravenous Delivery of Gemcitabine-Loaded Nanoparticles

More reduction of tumor volume following intravenous delivery of gemcitabine using unmodified CA nanoparticles in comparison to free drugs could be due to the influence of CA nanoparticles in altering the biodistribution of the loaded drugs, preventing homogeneous tissue distribution and accelerating tumor uptake of the drugs. The fascinating effect of the surface-modified, drug-encapsulated nanoparticles in dramatically regressing the tumor size could be due to more selective accumulation of the drugs into the tumor tissues, by preventing surface binding of serum proteins (i.e., opsonins) and consequential internalization of the nanoparticles by macrophages.

## 5. Conclusions

Non-covalent surface modification of CA nanoparticles with PEG is a promising approach to overcome the aggregation issue of hydrophobic drugs-loaded apatite particles, for efficient and targeted delivery of both hydrophilic and hydrophobic drugs, while retaining the pH-sensitivity behavior of the particles, a prerequisite for intracellular drug release during endosomal acidification. Surface modification has enabled enhancing in vitro cytotoxicity as well as in vivo tumor regression by reducing particle size and accelerating uptake by tumor cells. Our findings thus highlight the importance of carrying out further studies on PEGylated drug-loaded CA nanoparticles as one of the promising solubility enhancement strategies for drug delivery in preclinical cancer models.

## Figures and Tables

**Table 1:** Experimental conditions maintained for HPLC experiments for gemcitabine.

**Solvent**	H_2_O
**Mobile Phase**	70:30 ACN:H_2_O
**Flow Rate**	1 mL/min
**Column Temp**	30° C
**Detection**	DAD: 254 nm
**Run Time**	10 min
**Retention time**	2.2 min

**Table 2:** Experimental condition maintained for HPLC experiments for anastrozole.

**Solvent**	DMSO
**Mobile Phase**	40:60 ACN:MiliQ
**Flow Rate**	1 mL/min
**Column Temp**	30° C
**Detection**	DAD: 215 nm
**Run Time**	10 min
**Retention time**	4.8 min

**Table 3:** Enhancement of cytotoxicity (%) for gemcitabine-loaded CA particles in MCF7.

	100 pM	1 nM	10 nM	100 nM	1 µM
Uncoated	1.4 ± 1.8	2.8 ± 2	7.9 ± 1.8	10.8 ± 1.6	17.65 ± 2.7
Coated	1.9 ± 1.2	3.1 ± 1.1	9.2 ± 1.4	12.1 ± 1.9	20.4 ± 2.4

**Table 4:** Enhancement of cytotoxicity (%) for gemcitabine-loaded CA in 4T1.

	100 pM	1 nM	10 nM	100 nM	1 µM
Uncoated	1 ± 1.8	3.2 ± 1.2	3.5 ± 1.7	5.7 ± 2	10.4 ± 1.3
Coated	1.5 ± 1.9	3.8 ± 1.1	3.6 ± 1.15	8.5 ± 1.6	11.7 ± 1.5

**Table 5:** Enhancement of cytotoxicity (%) for anastrozole-loaded CA in MCF7.

	100 pM	1 nM	10 nM	100 nM	1 µM
Uncoated	1.1 ± 2.5	1.34 ± 2	4.2 ± 1.4	5.04 ± 2.2	0.8 ± 1.7
Coated	1.8 ± 1.4	3.7 ± 2.3	6.1 ± 2.4	7.2 ± 2.3	3 ± 1.9

**Table 6:** Enhancement of cytotoxicity (%) for anastrozole-loaded CA in 4T1.

	100 pM	1 nM	10 nM	100 nM	1 µM
Uncoated	1.6 ± 1.5	2 ± 2.4	6.3 ± 2	11.5 ± 2.7	8.7 ± 3.2
Coated	2.2 ± 2	4.7 ± 1.6	8.8 ± 2.25	18.4 ± 2.1	10.6 ± 3.76

**Table 7:** Estimation of gemcitabine and anastrozole binding affinity to CA particles in three different drug concentrations (20, 60 and 100 μM).

Drug Binding Affinity to CA			
Drug	20 µM	60 µm	100 µM
Gemcitabine	16% ± 0.25%	11.33% ± 0.87%	15.16% ± 0.1%
Anastrozole	0	0.05 ± 3	0

**Table 8:** Time-dependent cellular uptake of surface-modified and unmodified, gemcitabine-loaded CA particles in MCF7 cell line. CA was formed with different Ca^2+^ concentrations (7, 8 and 9 mM) and 20 μM of gemcitabine. Free gemcitabine (20 μM) was used as control.

20 uM	Free Gemcitabine	CA (Ca^2+^ 7 mM)	Coated CA (Ca^2+^ 7 mM)	CA (Ca^2+^ 8 mM)	Coated CA (Ca^2+^ 8 mM)	CA (Ca^2+^ 9 mM)	Coated CA (Ca^2+^ 9 mM)
1 h	30.5% ± 1.2%	52% ± 2%	57% ± 1%	55.5% ± 3.52%	57.1% ± 1.06%	60% ± 2%	61.2% ± 1.3%
4 h	50.75% ± 2.3%	79% ± 3.42%	86.65% ± 2%	80.8% ± 2.48%	83.66% ± 1.6%	81.2% ± 4.5%	84% ± 2.4%
24 h	100%	100%	100%	100%	100%	96,4% ± 2.5%	95.4% ± 3.2%

**Table 9:** Time-dependent cellular uptake of surface-modified and unmodified, anastrozole-loaded CA particles in MCF7 cell line. CA was formed with different Ca^2+^ concentrations (7, 8 and 9 mM) and 20 μM of anastrozole. Free anastrozole (20 μM) was used as control.

20 uM	Free Anastrozole	CA (Ca^2+^ 7 mM)	Coated CA (Ca^2+^ 7 mM)	CA (Ca^2+^ 8 mM)	Coated CA (Ca^2+^ 8 mM)	CA (Ca^2+^ 9 mM)	Coated CA (Ca^2+^ 9 mM)
1 h	18.5% ± 2.4%	30% ± 2.4%	51.% ± 2%	16.5% ± 1.4%	28.24% ± 2.7%	11.3% ± 1.82%	33.4% ± 2.76%
4 h	47.75% ± 2.3%	51% ± 1.7%	84.3% ± 1.6%	43.2% ± 2.43%	56.35% ± 1.8%	31.5% ± 2.6%	48.2% ± 1.6%
24 h	89.5% ± 3%	93% ± 2%	100%%	75.4% ± 2.6%	81% ± 1.6%	71.42% ± 1.8%	73.45% ± 2.2%
